# FGF21 promotes ischaemic angiogenesis and endothelial progenitor cells function under diabetic conditions in an AMPK/NAD+‐dependent manner

**DOI:** 10.1111/jcmm.16369

**Published:** 2021-02-17

**Authors:** Qiaoxia Dai, Xia Fan, Xue Meng, Shiyue Sun, Yue Su, Xiao Ling, Xiangjuan Chen, Kai Wang, Xiaozhen Dai, Chi Zhang, Sun Da, Guigui Zhang, Chunjie Gu, Hui Chen, Junhong He, Haiqi Hu, Lechu Yu, Xiaohong Pan, Yi Tan, Xiaoqing Yan

**Affiliations:** ^1^ Chinese‐American Research Institute for Diabetic Complications Department of Pharmacy Wenzhou Medical University Wenzhou China; ^2^ The Second School of Medicine Wenzhou Medical University Wenzhou China; ^3^ Department of Pharmacy The People's Hospital of YuHuan Taizhou China; ^4^ Department of Obstetrics and Gynecology The First Affiliated Hospital of Wenzhou Medical University Wenzhou China; ^5^ The First Affiliated Hospital of Wenzhou Medical University Wenzhou China; ^6^ School of Biomedicine Chengdu Medical College Chengdu China; ^7^ Ruian Center of Chinese‐American Research Institute for Diabetic Complications the Third Affiliated Hospital of Wenzhou Medical University Wenzhou China; ^8^ Institute of Life Sciences Wenzhou University Wenzhou China; ^9^ Department of Pharmacy Jinhua Municipal Central Hospital Jinhua China

**Keywords:** AMPK, diabetes, endothelial progenitor cells, fibroblast growth factor 21, NAD^+^

## Abstract

Diabetic vascular complications are closely associated with long‐term vascular dysfunction and poor neovascularization. Endothelial progenitor cells (EPCs) play pivotal roles in maintaining vascular homeostasis and triggering angiogenesis, and EPC dysfunction contributes to defective angiogenesis and resultant diabetic vascular complications. Fibroblast growth factor 21 (FGF21) has received substantial attention as a potential therapeutic agent for diabetes via regulating glucose and lipid metabolism. However, the effects of FGF21 on diabetic vascular complications remain unclear. In the present study, the in vivo results showed that FGF21 efficiently improved blood perfusion and ischaemic angiogenesis in both type 1 and type 2 diabetic mice, and these effects were accompanied by enhanced EPC mobilization and infiltration into ischaemic muscle tissues and increases in plasma stromal cell–derived factor‐1 concentration. The in vitro results revealed that FGF21 directly prevented EPC damage induced by high glucose, and the mechanistic studies demonstrated that nicotinamide adenine dinucleotide (NAD^+^) was dramatically decreased in EPCs challenged with high glucose, whereas FGF21 treatment significantly increased NAD^+^ content in an AMPK‐dependent manner, resulting in improved angiogenic capability of EPCs. These results indicate that FGF21 promotes ischaemic angiogenesis and the angiogenic ability of EPCs under diabetic conditions by activating the AMPK/NAD^+^ pathway.

## 1 INTRODUCTION

In parallel with the prevalence of the diabetes pandemic, diabetic vascular complications occur in the majority of patients. Most of the complications caused by diabetes are closely associated with long‐term vascular dysfunction and/or poor neovascularization^1^; therefore, promoting angiogenesis is considered a potential therapeutic strategy. Endothelial progenitor cells (EPCs) are the precursors of endothelial cells and contribute to vascular homeostasis and compensatory angiogenesis.^2^ However, the number of EPCs in diabetic patients is decreased, and diabetic EPCs display functional impairment, such as reduced proliferation, adhesion, migration, and incorporation into tubular structures.^3^ Therefore, efficient therapeutic strategies that can simultaneously promote EPC mobilization and improve EPC function with pharmacological agents are urgently needed for the treatment of diabetic patients with vascular complications.

Fibroblast growth factor 21 (FGF21) is a paracrine member of the FGF family that is able to regulate glucose and lipid metabolism.^4^ FGF21 stimulates glucose uptake by adipocytes^5^ and up‐regulates thermogenic activity.^6^ FGF21 can also promote lipolysis in adipocytes in response to starvation^7^ and enhance the expression and secretion of adiponectin from adipocytes, which further improves fatty acid oxidation and lipid clearance in the liver and skeletal muscle.^8^ In addition, FGF21 has insulin‐sensitizing ability^8^ and can ameliorate glucose tolerance^9^ by reducing glucose production in hepatocytes and stimulating glucose uptake in adipocytes. Moreover, FGF21 does not induce mitogenicity, hypoglycaemia or weight gain at any dose tested preliminarily in diabetic or healthy animals or when overexpressed in transgenic mice.^10^ Considering its multiple metabolic benefits, a large number of clinical trials have been registered to evaluate the therapeutic efficacy of several human FGF21 analogues for the treatments of type 2 diabetes (T2DM), dyslipidaemia and non‐alcoholic fatty liver disease (NAFLD).^11^

FGF21 also showed potential for treating cardiovascular complications of diabetes. Our previous study demonstrated that FGF21‐knockout (FGF21‐KO) mice are more prone to develop diabetic cardiomyopathy,^12^ which can be reversed by the administration of recombinant human FGF21.^13^ Moreover, Lin and colleagues^14^ found that FGF21 can prevent atherosclerosis, and our previous study showed that FGF21 deletion aggravates diabetes‐induced pathogenic changes in the aorta.^15^ However, the effect of FGF21 on diabetic ischaemic angiogenesis has not been characterized. In the present study, we investigated the therapeutic effects of FGF21 on ischaemic angiogenesis and blood reperfusion under diabetic conditions and uncovered the underlying mechanisms.

## 2 MATERIALS AND METHODS

### 2.1 Animals

Type 1 and type 2 diabetic mouse models were used in the present study. To induce a type 1 diabetes (T1DM) model, male C57BL/6 mice (GemPharmatech) aged 8‐10 weeks received multiple low‐dose streptozotocin (STZ; Sigma) via intraperitoneal injection (50 mg/kg body weight, once daily for 5 consecutive days). Mice with blood glucose >13.8 mmol/L on the 7th day after the last injection were considered to be T1DM models, and then, these mice were maintained for 2 more months before receiving hind limb ischaemia (HLI) surgery. Three‐month‐old male *db/db* mice (GemPharmatech) were used as the T2DM models. All animal procedures were approved by the Animal Policy and Welfare Committee of Wenzhou Medical University.

### 2.2 HLI models and FGF21 administration

Both T1DM and T2DM mice were randomly assigned into two groups, the control group and the FGF21 group (n = 10‐15/group), and received HLI surgery as described in our previous study.^16^ In brief, after sufficient anaesthesia with isoflurane, the right hind limb was dissected. The superficial femoral artery was double‐ligated with 6‐0 silk sutures, cut off and excised with an electrical coagulator (Fine Science Tools). Then, the overlying skin was closed with 4‐0 silk sutures. The FGF21 group was pre‐treated with FGF21 (0.5 mg/kg, daily) via intraperitoneal injection for 2 days before HLI surgery and continually treated with FGF21 for an additional 3 or 28 days after surgery until the mice were killed. The mice in the control group were treated with vehicle phosphate‐buffered saline (PBS). Four mice from each group were killed at 3rd day after surgery, and the remaining mice were killed at 28th day after surgery. Blood and gastrocnemius muscle samples were collected for further assays.

### 2.3 Blood flow perfusion imaging

Blood flow perfusion was measured before surgery and at days 0, 3, 7 14, 21, 28 after surgery using a PeriCam perfusion speckle imager (PSI, Perimed). Non‐ischaemic hind limb was used as self‐control, and blood flow perfusion was presented as the percentage of blood flow in the ischaemic limb (right) relative to blood flow in the normal limb (left).

### 2.4 Immunofluorescence staining

The extent of angiogenesis was assessed by measuring capillary density through CD31 and dystrophin (to indicate myofiber) staining. Briefly, frozen sections (6 μm) of ischaemic gastrocnemius muscles dissected from ischaemic hind limbs at 28th day post‐HLI surgery were fixed with cold methanol for 15 minutes. After 3 washes with PBS, the sections were incubated with blocking buffer (PBS containing 5% goat serum) for 1 hour. Thereafter, the sections were incubated overnight with primary antibody against dystrophin (Proteintech) at 4°C. After 3 washes with PBS, the sections were incubated with corresponding PE‐conjugated secondary antibody (Cell Signaling Technology) and FITC‐conjugated anti‐CD31 primary antibody (BD Biosciences) in the dark at room temperature for 1 hour. After 3 washes with PBS, the sections were sealed with antifade reagent, and pictures were taken using fluorescence microscope (Olympus IX71; Olympus). Capillary density was expressed as the number of CD31‐positive capillaries per myofiber. Circulating EPCs infiltrating ischaemic muscle at 3rd day post‐HLI surgery were recognized by CD34 and VEGFR2 staining, and the expression of FGFR1 and β‐klotho (KLB) on EPCs was also detected.

### 2.5 Flow cytometry assay

At 3rd day after surgery, circulating EPCs were detected by flow cytometry as described in our previous study.^17^ Briefly, after sufficient anaesthesia with 1% pentobarbital sodium, peripheral blood was collected in a lithium heparin tube (BD, Franklin). 100 mL anticoagulant blood was incubated with FITC‐conjugated antimouse CD34 (Biolegend) and PE‐conjugated antimouse VEGFR2 (Biolegend) antibodies in the dark at 4°C for 30 minutes, followed by blood cell lysis using red cell lysis buffer (Cell Signaling Technology). After washing twice with PBS, the cells were resuspended with 400 μL PBS, and CD34^+^/VEGFR2^+^ EPCs were analysed by flow cytometry (Agilent).

### 2.6 Plasma stromal cell–derived factor‐1 (SDF‐1) detection

Anticoagulant blood was centrifuged at 2000 rpm at 4°C for 20 minutes, and plasma was collected for SDF‐1 detection using an SDF‐1 Quantikine enzyme‐linked immunosorbent assay (ELISA) kit (R&D Systems) according to the manufacturer's instructions.

### 2.7 Human umbilical cord blood EPCs isolation and identification

Human umbilical cord blood samples (20‐40 mL) from healthy newborns were collected and anticoagulated with citrate phosphate dextrose (CPD) solution. The research ethics committee of the First Affiliated Hospital of Wenzhou Medical University approved all protocols, and informed consent was obtained from the parents of the newborns. EPCs were isolated as described in previous studies.^17^, ^18^, ^19^ Briefly, cord blood was diluted 1:1 with Hanks' balanced salt solution (HBSS; Invitrogen), carefully overlaid onto an equivalent volume of Histopaque 1077 (Sigma) and centrifuged at 400 *g* for 30 minutes at room temperature. Thereafter, the buffy coat was collected and washed with HBSS twice, and cell aggregate was resuspended in EGM‐2 (Lonza) supplemented with 2% FBS (Sigma) and plated onto 6‐well plates that were pre‐coated with human fibronectin (2 μg/cm^2^; BD Biosciences). After culturing for 24 hours, unattached cells and debris were removed by washing with EGM‐2 culture medium. The medium was changed daily for 7 days and thereafter on alternate days. Clones appeared between day 14‐21 and reached 80% confluence on approximately day 28. After subculturing, the cell surface antigens CD34, VE‐cadherin (CD144), VEGFR2, CD14 and CD45 (BD Biosciences) were detected by flow cytometry assay to characterize the EPCs. The EPCs were expanded to the fourth or fifth passage for further analysis.

### 2.8 RNA interference

To knockdown nicotinamide phosphoribosyltransferase (NAMPT) or Sirtuin1 (Sirt1)‐Sirt7 expression in EPCs, small‐interfering RNAs (siRNAs) against human NAMPT or Sirt1‐Sirt7 (Table S1), purchased from GenePharma, were transfected into EPCs using Lipofectamine 3000 (Thermo Fisher) according to the manufacturer's instructions. After transfection for 48 hours, the expression of NAMPT or Sirt1 was determined by quantitative real‐time PCR (qRT‐PCR).

### 2.9 qRT‐PCR

Total mRNA was extracted from each group of EPCs using an RNA extraction kit (Tiangen) and reverse‐transcribed to cDNA using a high‐capacity cDNA reverse transcription kit (Thermo Fisher Scientific). qRT‐PCR was performed using a SYBR Green PCR Master Mix kit (Invitrogen) according to the manufacturer's instructions on a 7500 Real‐Time PCR machine (Applied Biosystems). The specific primers for human FGFR1‐FGFR4, Sirt1‐Sirt7, and NAMPT (Table S2) were purchased from GenScript. β‐Actin was used as an internal loading control.

### 2.10 Cell scratch recovery assay

Cell migration was investigated by a cell scratch recovery assay. EPCs (1 × 10^5^/well) were seeded onto 24‐well plates and maintained to confluence. Thereafter, a scratch was made in the wells using pipette tips. After washing away cell debris with PBS, the remaining cells were cultured in MCDB131 medium containing 27.5 mmol/L mannitol (Man) or 27.5 mmol/L glucose (high glucose, HG), along with 200 ng/mL FGF21 or an equivalent volume of PBS, and incubated at 37°C in a humidified environment with 5% CO_2_ for 24 hours. Mitomycin (1 μmol/L; Sigma) was used to exclude the influence of cell proliferation. Then, the scratches were recorded under a light microscope (Leica DMI3000B; Leica) equipped with a digital camera (Olympus DP25). The recovery of the scratches was measured using ImageJ (http://rsbweb.nih.gov/ij/).

### 2.11 Matrigel tube formation assay

A tube formation assay was conducted as described previously^20^ with slight modification. Briefly, EPCs were treated as in the cell scratch recovery assay and thereafter trypsinized and resuspended. Growth factor‐reduced Matrigel (BD Biosciences) was thawed at 4°C overnight, and then, 10 μL Matrigel was added to a μ‐slide (Ibidi) and incubated at 37°C for 30 minutes to polymerize. Resuspended EPCs were seeded onto the Matrigel, and tube‐like structures were recorded 8 hours later under a light microscope (Leica) equipped with a digital camera (Olympus DP25). The length of tube‐like structures in the images was measured using ImageJ software (http://rsbweb.nih.gov/ij/).

### 2.12 NAD^+^ content assay

Nicotinamide adenine dinucleotide (NAD^+^) content was determined with NAD/NADH Assay kit (Abcam) according to the manufacturer's instructions. NAD^+^ content was normalized by protein concentration.

### 2.13 β‐Galactosidase staining

β‐Galactosidase was assayed using a senescence β‐galactosidase staining kit (Beyotime) according to the manufacturer's instructions. In brief, EPCs were treated as described above, and after removing the cell culture medium and washing the plate once with PBS, the EPCs were fixed for 15 minutes at room temperature with 1 mL of fixative. Thereafter, the cells were incubated with staining working solution overnight at 37°C, and after 3 washes with PBS, pictures were taken.

### 2.14 Western blot

EPCs were washed twice with pre‐cooled PBS and lysed in RIPA buffer solution (Cell Signaling Technology) containing phosphatase inhibitor and protease inhibitor (Roche) for 15‐30 minutes on ice. After determining the protein concentration with Quick Start Bradford Dye Reagent (Bio‐Rad), proteins were separated on a 10% SDS‐PAGE and transferred onto nitrocellulose membranes (Bio‐Rad).^17^ After blocking in 5% skim milk for 1 hour, the membranes were incubated overnight at 4°C with antibodies against phospho‐extracellular regulated protein kinases 1/2 (p‐Erk1/2), Erk1/2, CD38, NAMPT, poly (ADP‐ribose) polymerase (PARP), CD31, phospho‐adenosine 5’‐monophosphate (AMP)‐activated protein kinase (p‐AMPK), AMPK, glyceraldehyde‐3‐phosphate dehydrogenase (GAPDH) or β‐actin (Cell Signaling Technology). After 3 washes with Tris‐buffered saline with Tween‐20 (TBST), the membranes were incubated with the corresponding HRP‐conjugated secondary antibody (Cell Signaling Technology) at room temperature for 1 hour. The bands were visualized using ECL and detected by a Western blot imaging system (Tanon) after 3 washes with TBST.

### 2.15 Statistics

The results are based on at least three independent experiments and expressed as the means ± SD. Statistical analysis was performed using GraphPad Prism 8 software (GraphPad) by one‐way ANOVA and Student's *t* test as appropriate. Significance was considered to be indicated by a *P*‐value less than 0.05.

## 3 RESULTS

### 3.1 FGF21 improves post‐HLI blood perfusion and neovascularization in T2DM

**FIGURE 1:**
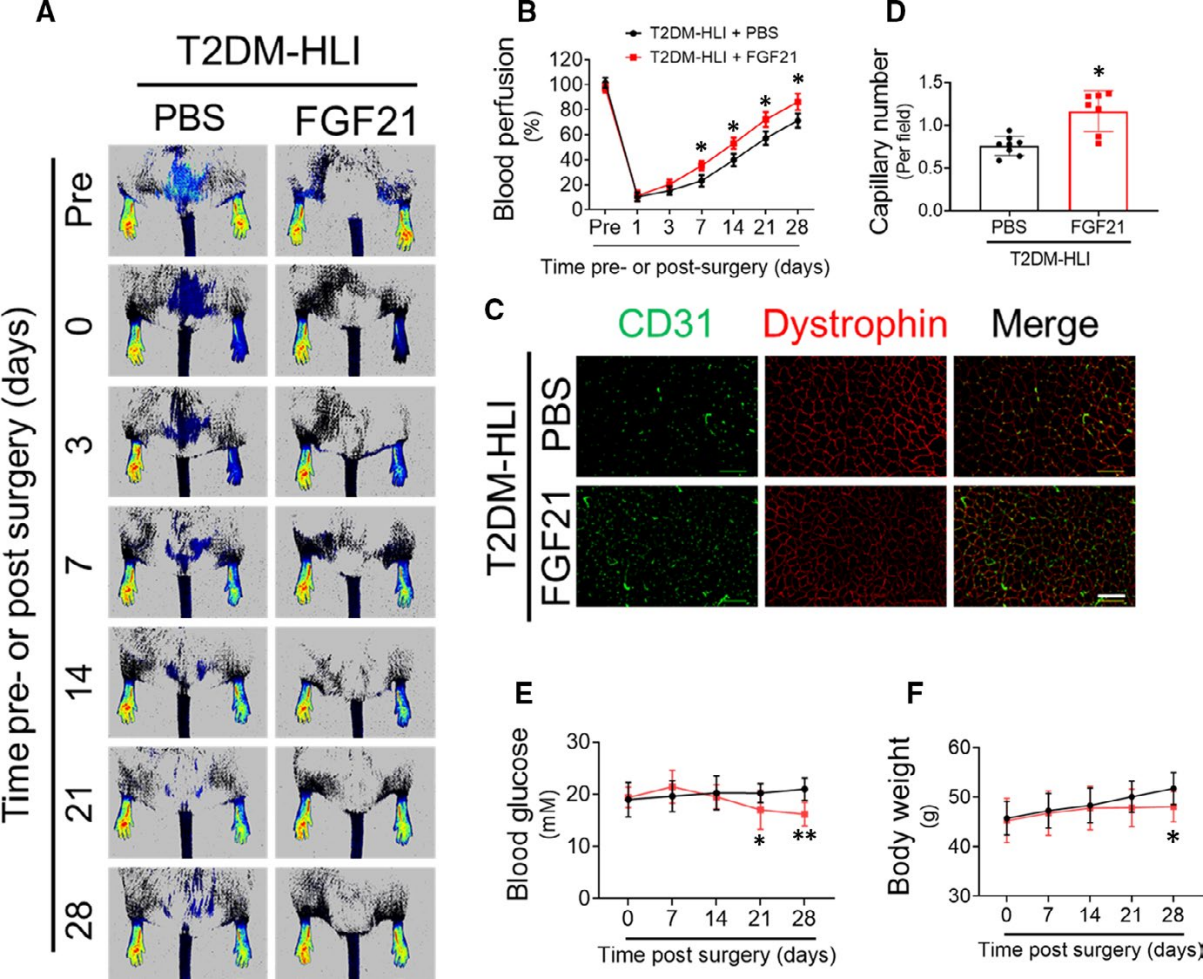
FGF21 improves blood perfusion and angiogenesis in type 2 diabetic hind limb ischaemia (HLI) model. The proangiogenic effects of FGF21 in HLI were investigated in *db/db* type 2 diabetic mice. (A) The blood perfusion analysis using a PeriCam PSI before and after HLI surgery with or without FGF21 (500 μg/kg bodyweight, daily) treatment. (B) Blood perfusion was quantified by ImageJ as the ratio of ischaemic perfusion to non‐ischaemic limb perfusion. Immunofluorescent staining (C) and quantification (D) of CD31‐positive capillaries in transverse sections of gastrocnemius muscle tissue from ischaemic hind limbs at 28th day after HLI surgery. Capillary density is presented as the number of CD31‐positive capillaries per muscle fibre as identified by dystrophin staining. Blood glucose (E) and bodyweight (F) were monitored before and after HLI surgery. n = 7‐8 mice per group. The data shown in the graphs represent the means ± SD *P < 0.05 vs the PBS group, **P < 0.01 vs the T2DM‐HLI + PBS group. Bar = 100 μm. PBS, phosphate‐buffered saline; PSI, perfusion speckle imager; T2DM, type 2 diabetes mellitus

To investigate whether FGF21 has therapeutic effects on diabetic ischaemia, HLI was induced in *db/db* mice, and blood perfusion was detected using PSI. The results showed that blood perfusion in the ischaemic hind limbs of the FGF21‐treated mice was obviously higher than that of the PBS‐treated mice since day 7 post‐HLI surgery (Figure 1A,B), indicating that FGF21 can improve blood perfusion in ischaemic hind limbs under T2DM conditions. To further assess the effect of FGF21 on ischaemic angiogenesis, capillary density in ischaemic gastrocnemius muscle was determined by CD31 staining, and the results showed that the FGF21‐treated mice had higher capillary density in their ischaemic muscle than the PBS‐treated mice (Figure 1C,D).

FGF21 is an important regulator of metabolism and was reported to ameliorate abnormal glucose and lipid metabolism in T2DM.^11^ As expected, FGF21 administration lowered blood glucose levels (Figure 1E) and bodyweight (Figure 1F) in the present study. However, the beneficial effects of FGF21 on enhancing blood perfusion appeared earlier than that on lowering blood glucose or losing weight (the 7th day vs the 21th or 28th day after surgery), revealing that FGF21 improving diabetic ischaemia angiogenesis and blood perfusion may be, at least partly, independent of its effects on lowering blood glucose and bodyweight.

### 3.2 FGF21 improves post‐HLI blood perfusion, neovascularization and EPC mobilization in T1DM

**FIGURE 2:**
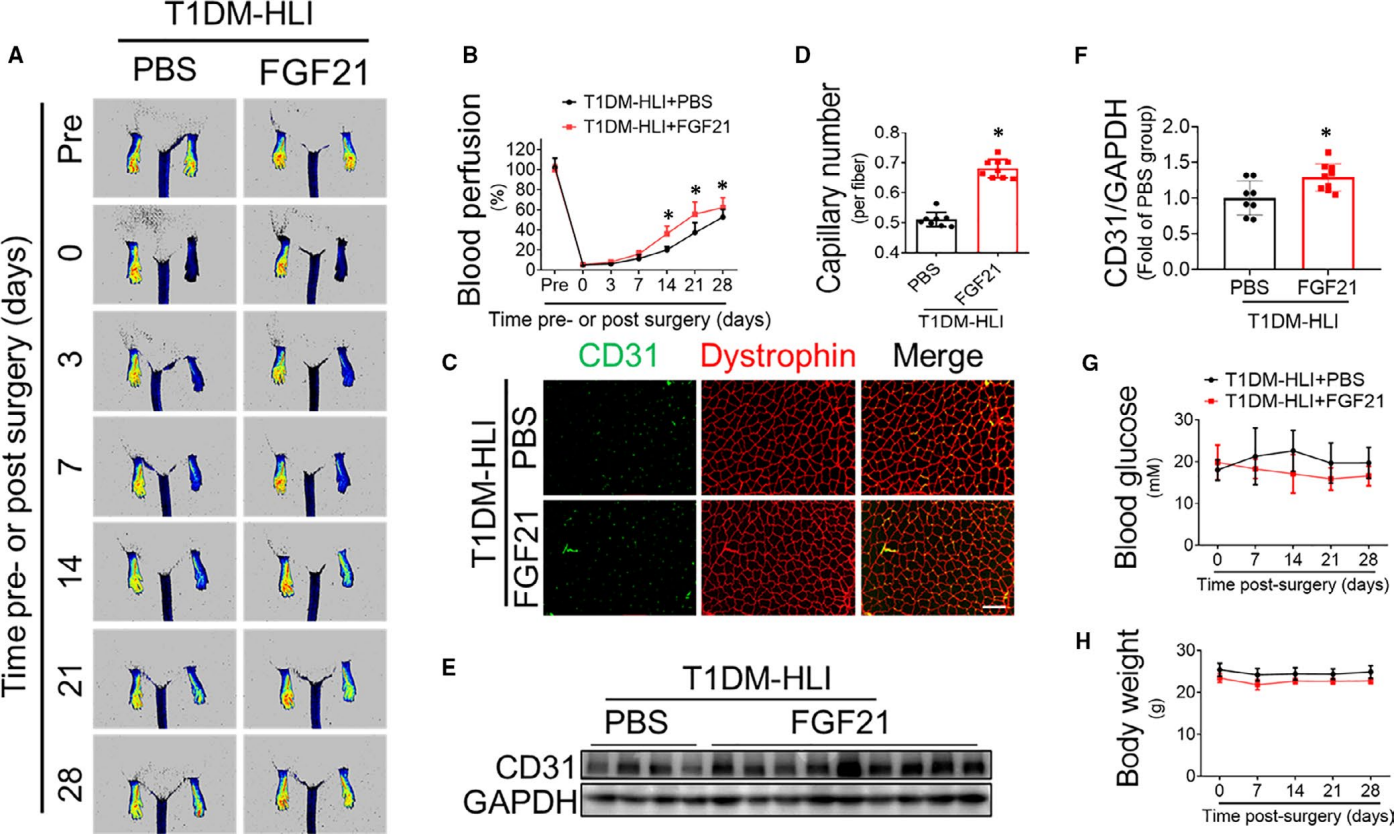
FGF21 improves blood perfusion and angiogenesis in HLI in STZ‐induced type 1 diabetes. The proangiogenic effects of FGF21 in HLI were investigated in STZ‐induced type 1 diabetic mice. (A) Blood perfusion analysis using a PeriCam PSI before and after HLI surgery with or without FGF21 treatment. (B) Blood perfusion was quantified by ImageJ as the ratio of ischaemic to non‐ischaemic limb perfusion. Immunofluorescent staining (C) and quantification (D) of CD31‐positive capillaries in transverse sections of gastrocnemius muscle tissue from ischaemic hind limbs at 28th day after HLI surgery. Capillary density is presented as the number of CD31‐positive capillaries per muscle fibre as identified by dystrophin staining. CD31 expression in ischaemic gastrocnemius muscle was also detected by Western blot analysis (E) and quantified (F), and glyceraldehyde 3‐phosphate dehydrogenase (GAPDH) was used as loading control. Blood glucose (G) and bodyweight (H) were monitored before and after HLI surgery. The data shown in the graphs represent the means ± SD n = 8‐9 mice per group. *P < 0.05 vs the PBS group. Bar = 100 μm. T1DM, type 1 diabetes mellitus

In line with the observation in the T2DM contexts, FGF21 treatment also significantly improved the blood perfusion in the ischaemic mouse tissues of compared with that in the PBS‐treated control mice since day 14 post‐HLI surgery (Figure 2A,B), which was accompanied by significantly higher CD31‐positive capillary staining (Figure 2C,D), and an apparent increase in CD31 protein expression (Figure 2E,F) in the ischaemic muscle of FGF21‐treated mice. In contrast, FGF21 administration did not affect blood glucose (Figure 2G) or bodyweight (Figure 2H) of the T1DM mice within 28 days after HLI induction, which further confirmed that FGF21 can enhance ischaemic angiogenesis and blood perfusion under diabetic conditions independent of its effect in lowering blood glucose and bodyweight.

**FIGURE 3:**
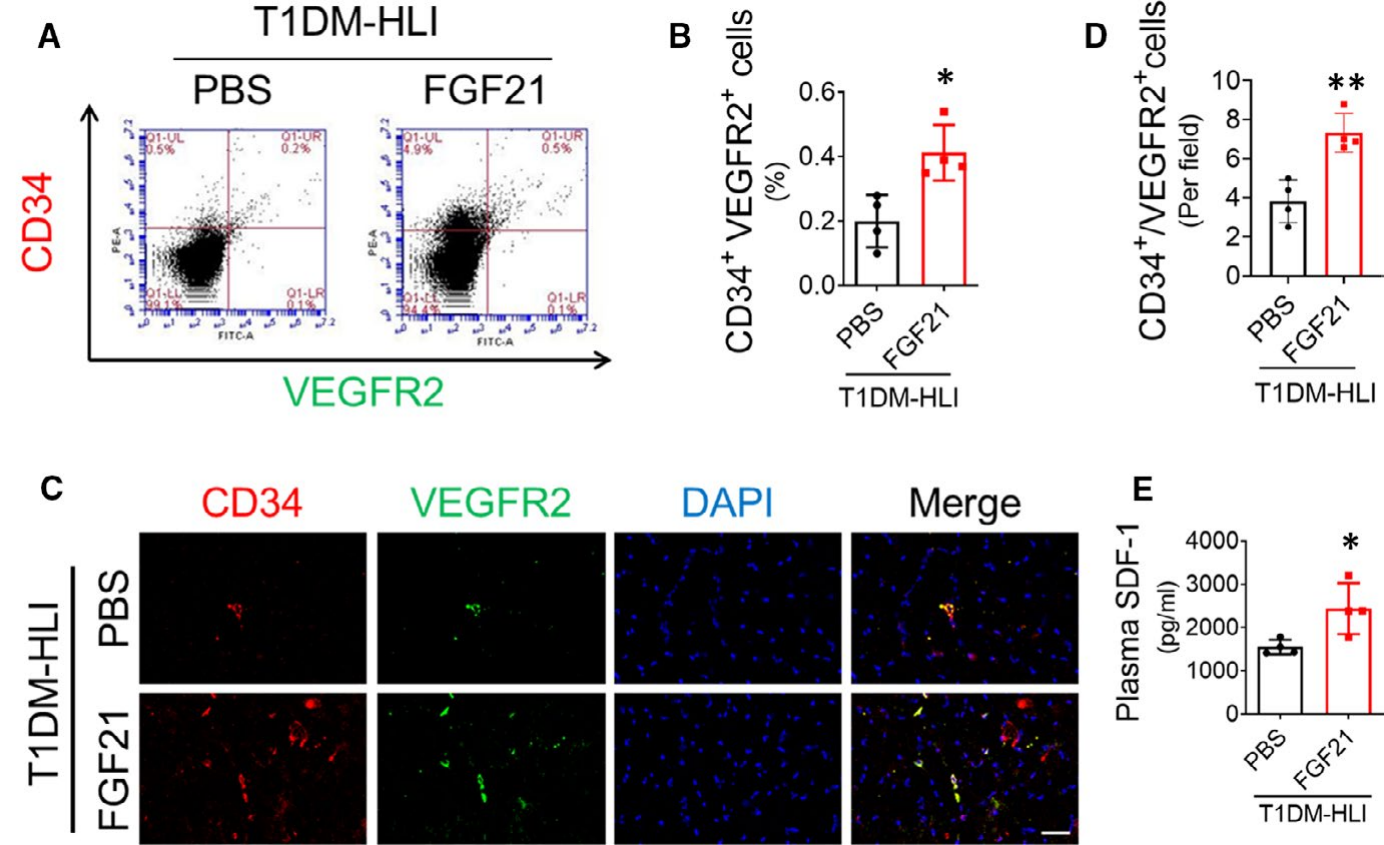
FGF21 improves EPC mobilization and incorporation into ischaemic tissue in STZ‐induced type 1 diabetes. At 3rd day after surgery, peripheral blood was collected to evaluate the percentage of EPCs (CD34^+^/VEGFR2^+^) in the peripheral blood by flow cytometry (A, B). CD34^+^/VEGFR2^+^ EPCs incorporating into gastrocnemius muscle tissue from ischaemic hind limbs at 3rd day after surgery were detected by immunofluorescent staining (C) and quantified (D), and nuclei were identified by 4',6‐diamidino‐2‐phenylindole (DAPI) staining. (E) The SDF‐1 concentration in plasma was detected by enzyme‐linked immunosorbent assay. n = 4 mice per group. The data shown in the graphs represent the means ± SD *P < 0.05 vs the PBS group. Bar = 50 μm. SDF‐1, stromal cell–derived factor‐1

EPCs trigger angiogenesis under physiological and pathological conditions.^2^ EPC mobilization is a pivotal step in EPC participation in angiogenesis and is impaired under diabetic conditions.^21^ In the present study, we found that FGF21 administration increased the number of CD34^+^/VEGFR2^+^ EPCs in the peripheral blood of STZ‐induced T1DM mice at day 3 post‐HLI (Figure 3A,B). The immunofluorescence staining results also showed that there was an increase in CD34^+^/VEGFR2^+^ EPCs residing in ischaemic gastrocnemius muscle (Figure 3C,D). These results demonstrated that FGF21 can also enhance EPC mobilization and infiltration into ischaemic tissue under diabetic conditions. SDF‐1 is one of the major chemokines that regulate EPC mobilization and homing to the site of neovascularization.^18^ The ELISA results showed that the plasma SDF‐1 concentration in the FGF21‐treated mice was higher than that in the PBS‐treated mice at day 3 post‐HLI (Figure 3E). The elevated SDF‐1 may contribute to enhanced EPC mobilization in the FGF21‐treated T1DM mice.

### 3.3 FGF21 ameliorates EPC dysfunction induced by high glucose

**FIGURE 4:**
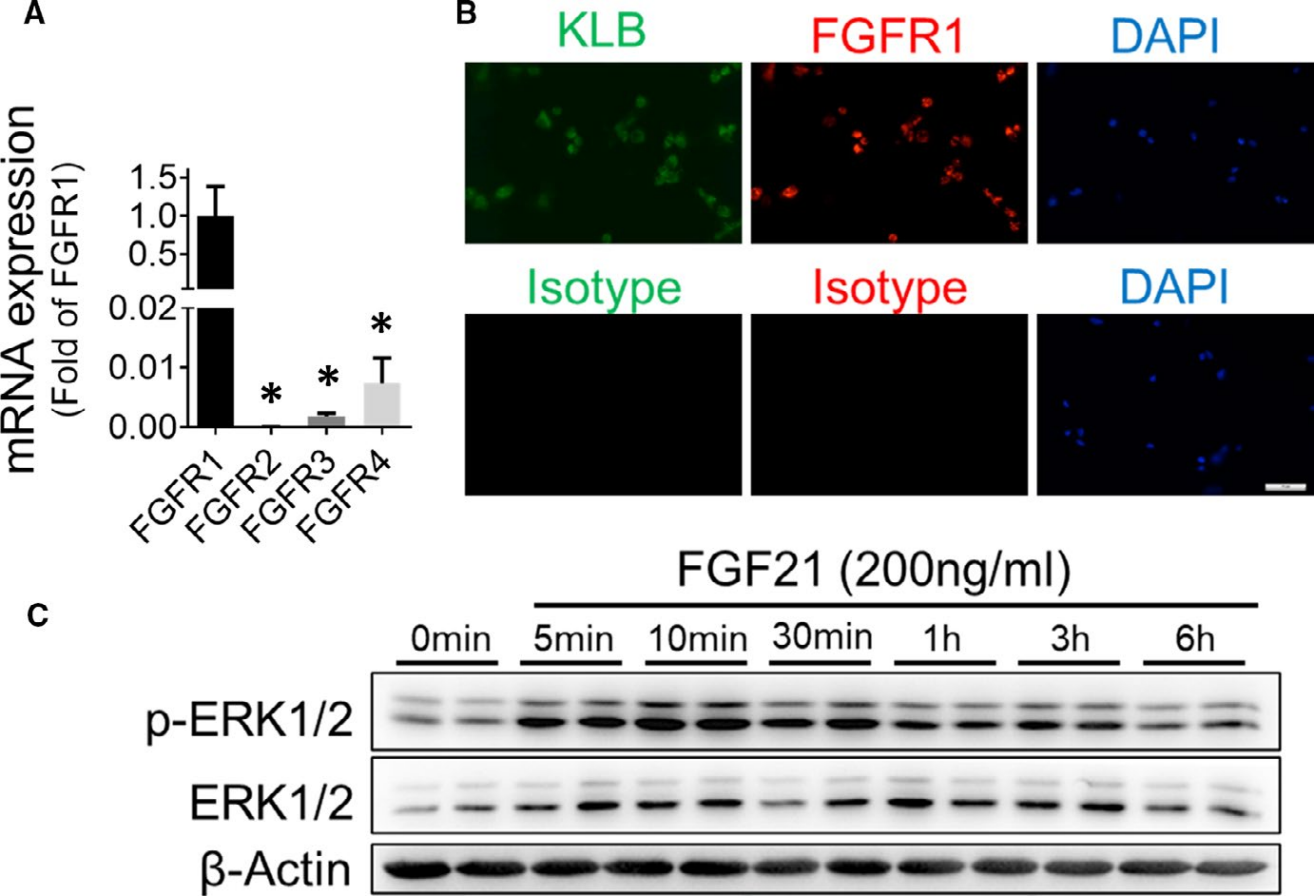
EPCs respond to FGF21 stimulation. A, The expression of fibroblast growth factors (FGFR1‐FGFR4) was detected by qRT‐PCR. n = 3 per group. B, FGFR1 and β‐klotho (KLB) expression on EPCs was detected by immunofluorescence staining, and nuclei were identified by DAPI staining. C, The time effect of extracellular signal‑regulated protein kinase (ERK1/2) phosphorylation in EPCs after FGF21 stimulation was detected by Western blot analysis. The data shown in the graphs represent the means ± SD *P < 0.05 vs the FGFR1. Bar = 50 μm

Considering the critical role of EPCs in angiogenesis and the capability of FGF21 to promote EPC mobilization in T1DM, we hypothesized that the FGF21 improvement of blood perfusion and angiogenesis in diabetic ischaemia may have a direct protective effect on diabetic EPC functions. To test this hypothesis, the EPCs were isolated from human cord blood as previously described^19^ and characterized by flow cytometry to distinguish EPCs, which are positive for CD144, CD34 and VEGFR2 and negative for CD14 or CD45 (Figure S1). As FGF21 exerts its biological functions by binding to its receptor FGFR1 and coreceptor KLB,^22^ FGF21 receptor expression on EPCs was investigated by qRT‐PCR. The results showed that FGFR1‐FGFR4 mRNAs were all detectable in the EPCs, and the expression of FGFR1 was much higher than that of any other FGFR (Figure 4A). The immunofluorescence staining results confirmed that both FGFR1 and KLB proteins are expressed on EPCs (Figure 4B), and treating EPCs with FGF21 stimulated the phosphorylation of the classic FGFR downstream signalling proteins ERK1/2^23^ in a time‐dependent manner (Figure 4C), demonstrating that FGF21 is a functional ligand for FGFRs and KLB on EPCs.

**FIGURE 5:**
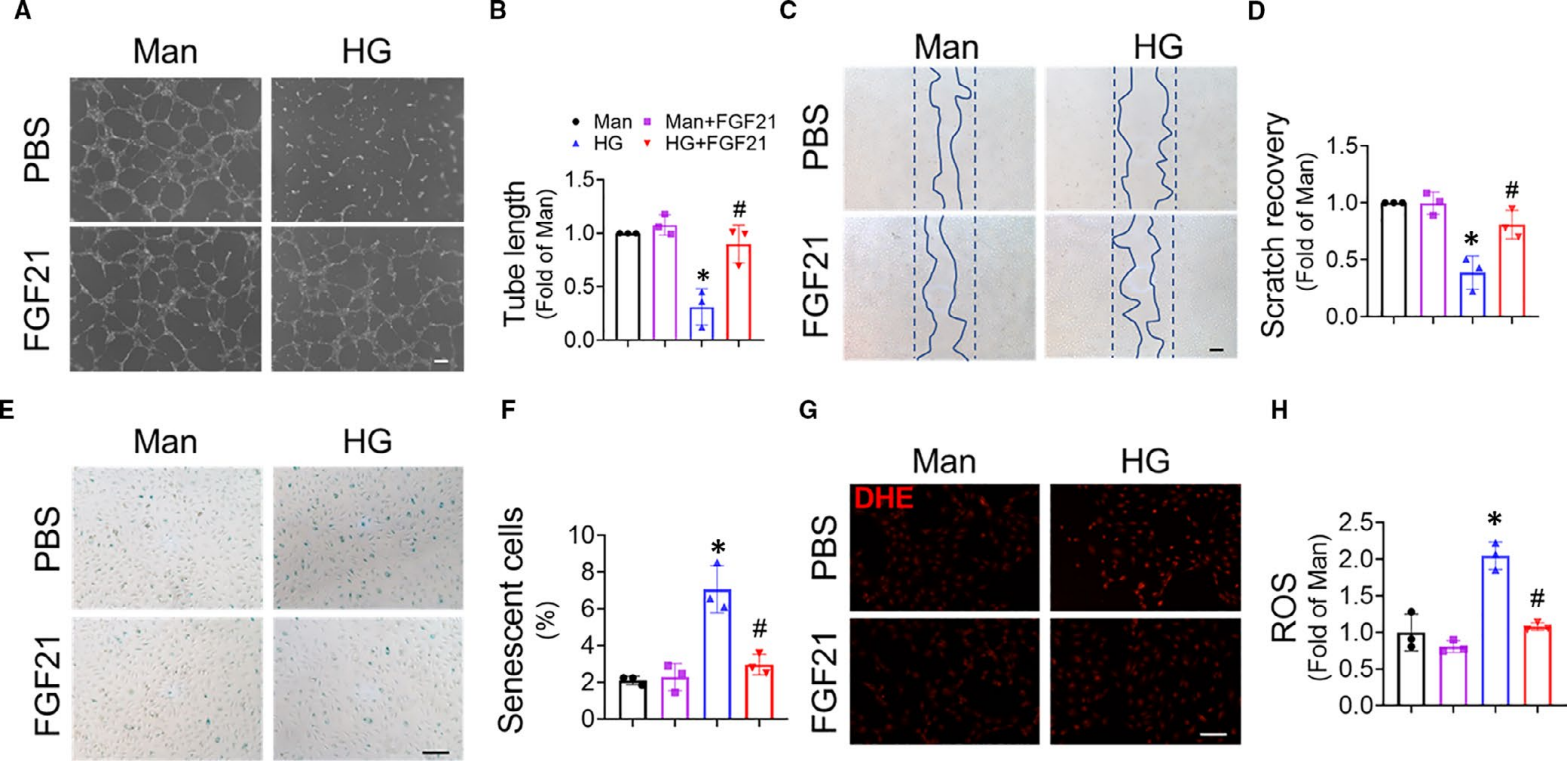
FGF21 improves the angiogenic function of EPCs impaired by high glucose. EPCs were treated with high glucose (HG) in the presence or absence of FGF21 for 24 h, and mannitol (Man) was used as an osmotic control. The effects of FGF21 on the angiogenic function of the EPCs were evaluated by Matrigel tube formation assay (A), and the tube length was quantified using ImageJ and normalized to that of the mannitol treatment group (B). The effects of FGF21 on EPC migration were evaluated by cell scratch assay (C), and scratch recovery was quantified using ImageJ and normalized to that of the mannitol treatment group (D). The effects of FGF21 on EPC senescence were evaluated by β‐galactosidase staining (E), and the percentage of β‐galactosidase‐positive cells was determined (F). The antioxidative effects of FGF21 were determined by fluorescent probe dihydroethidium (DHE) staining of reactive oxygen species (ROS) (G), and the fluorescence intensity of DHE was measured by a fluorescence microplate reader and normalized to that of the mannitol treatment group (H). Three independent experiments were performed for each study. The data shown in the graphs represent the means ± SD *P < 0.05 vs the mannitol group and ^#^ *P* < 0.05 vs the HG treatment group. Bar = 100 μm

To determine whether FGF21 can improve the angiogenic function of EPCs under diabetic conditions, EPCs were exposed to HG (MCDB131 containing 27.5 mmol/L glucose) to mimic hyperglycaemia in vivo. Matrigel tube formation assays showed that HG significantly impaired the tube formation capability of EPCs, which could be prevented by FGF21 treatment (Figure 5A,B). Cell scratch assay showed that HG significantly delayed scratch recovery, whereas FGF21 treatment significantly accelerated this process (Figure 5C,D). EPC senescence also occurred under diabetic conditions.^24^ The β‐galactosidase assay results showed that HG exacerbated EPC senescence, which was attenuated by FGF21 treatment (Figure 5E,F). Diabetes also induced oxidative stress in the EPCs,^17^ and the DHE staining results showed that HG significantly increased superoxide levels in EPCs, whereas FGF21 treatment decreased superoxide levels (Figure 5G,H). All these in vitro results demonstrated that FGF21 can exert direct influences on EPCs and improve the impaired proangiogenic function of EPCs under diabetic conditions.

### 3.4 FGF21 prevents HG‐induced EPC dysfunction via increasing NAD^+^ content

**FIGURE 6:**
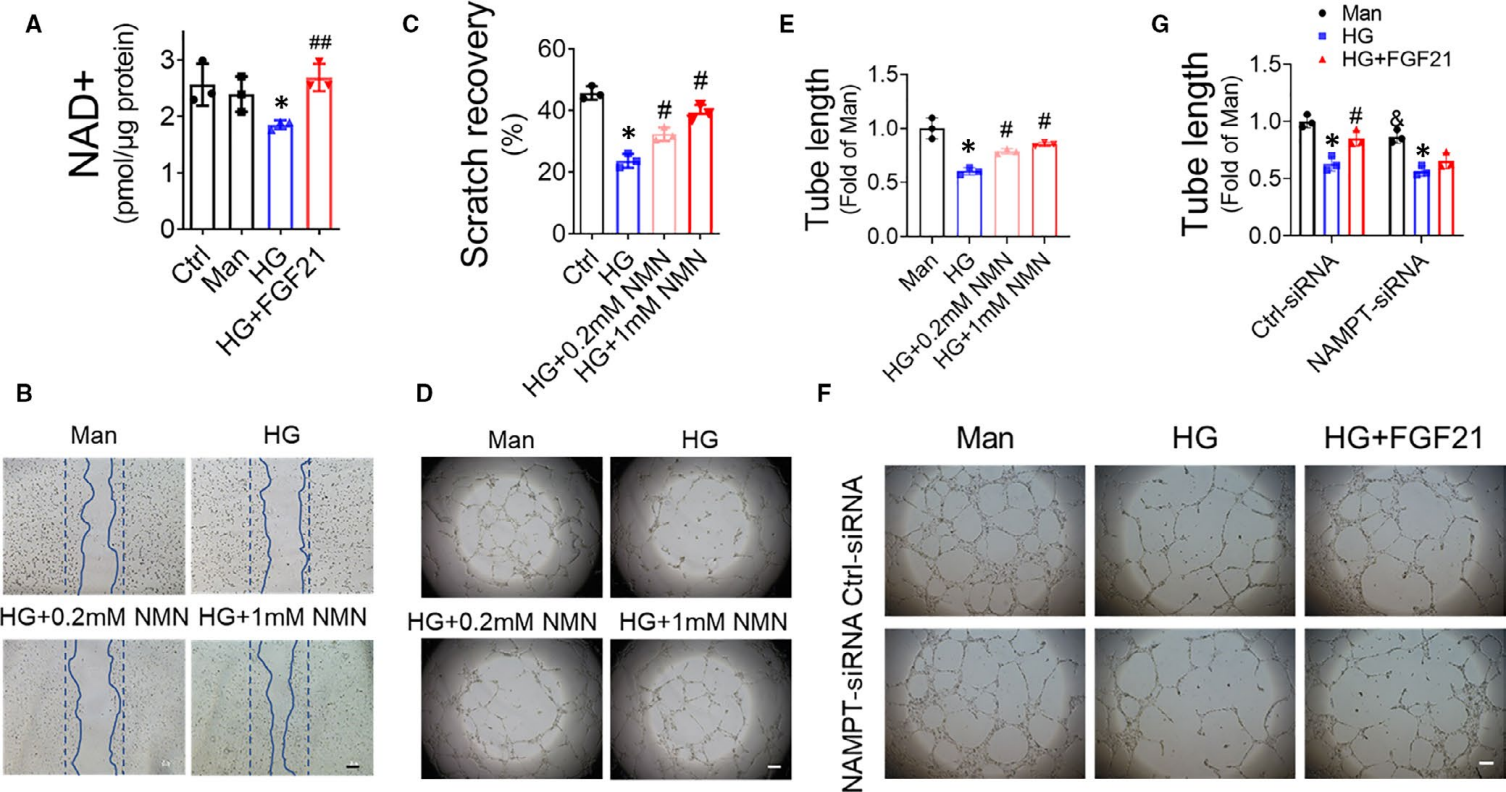
FGF21 prevents EPC dysfunction induced by HG by increasing NAD+ content. EPCs were treated with HG in the presence or absence of FGF21 for 24 h, and mannitol (Man) was used as an osmotic control. (A) The influence of FGF21 on NAD+ content in HG‐treated EPCs was determined using an NAD/NADH assay kit, and the NAD+ content was normalized by the protein amount. (B) The effects of NAD+ increased via supplementation with NMN on the angiogenic function of EPCs were evaluated by Matrigel tube formation assay, and (C) the tube length was quantified by Image J and normalized to that of the mannitol treatment group. (D) The effects of NMN on EPC migration were evaluated by cell scratch assay (D), and the scratch recovery was quantified using Image J and normalized to that of the mannitol treatment group (E). The effects of knocking down NAMPT on FGF21‐enhanced angiogenic function of HG‐treated EPCs were evaluated by Matrigel tube formation assay (F), and the tube length was quantified by ImageJ and normalized to the mannitol treatment group (G). Data shown in graphs represent the mean ± SD *P < 0.05 vs mannitol group; ^#^ *P* < 0.05 vs HG group; ^##^ *P* < 0.01 vs HG group; and ^&^ *P* < 0.05 vs HG Ctrl‐siRNA group. Bar = 10 μm

NAD^+^ plays a critical role in maintaining vascular function, and NAD^+^ deficiency results in EPC dysfunction under diabetic conditions.^21^ In the present study, NAD^+^ content was decreased in HG‐treated EPCs, a result that was reversed by FGF21 treatment (Figure 6A). To identify the protective role of NAD^+^ against EPC dysfunction under diabetic conditions, we investigated the effect of NAD^+^ supplement nicotinamide mononucleotide (NMN) on the migration and tube formation capability of the HG‐treated EPCs. Cell scratch assay showed that NMN improved HG‐treated EPC migration in a dose‐dependent manner (Figure 6B,C). Similarly, NMN also enhanced the tube formation capability of EPCs impaired by HG (Figure 6D,E). In contrast, knocking down NAMPT (Figure S2), the rate‐limiting enzyme for NAD^+^ biosynthesis, abolished the protective effect of FGF21 on tube formation of the HG‐treated EPCs (Figure 6F,G), which confirmed the critical role of increasing NAD^+^ content in FGF21 ameliorating HG‐induced EPC dysfunction.

The sirtuins (Sirt1‐Sirt7) are a family of NAD^+^‐dependent deacylases with remarkable abilities to prevent ageing and age‐related diseases.^25^ To identify which sirtuin member mediates the beneficial effects of NAD^+^ on EPCs, Sirt1 to Sirt7 were sequentially knocked down by the corresponding siRNAs (Figure S3A). The Matrigel tube formation assay showed that only Sirt1‐siRNA impaired the enhancing effect of NAD^+^ precursors on the tube formation capability of the HG‐treated EPCs (Figure S3B,C), confirming that NAD^+^ improves the angiogenic function of diabetic EPCs through Sirt1.

### 3.5 FGF21 increases NAD^+^ content by activating the AMPK pathway in EPCs

NAMPT,^26^ CD38^27^ and PARP^28^ are well documented to regulate NAD^+^ metabolism. To uncover the underlying mechanism by which FGF21 increases the NAD^+^ content of HG‐treated EPCs, the expression of NAMPT, CD38 and PARP was detected by Western blot. However, the results showed that the expression of NAMPT, CD38 and PARP was not changed by either HG or FGF21 treatment (Figure S4A,B), indicating that the FGF21‐stimulated elevation of NAD^+^ content in HG‐treated EPCs is not attributable to NAMPT, CD38 or PARP.

**FIGURE 7:**
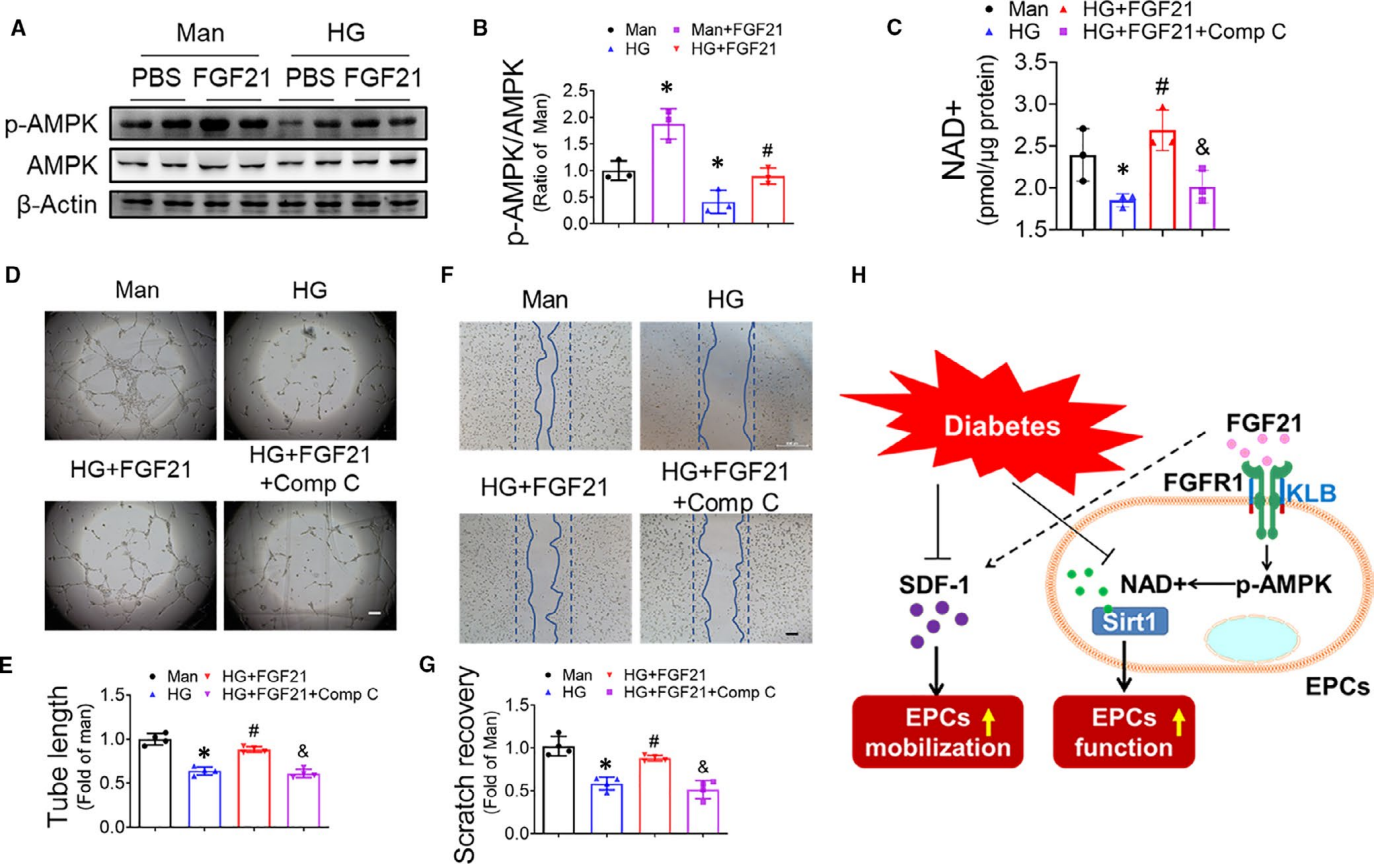
FGF21 increases NAD+ content by activating the AMPK pathway. EPCs were treated with HG in the presence or absence of FGF21 for 24 h, and mannitol (Man) was used as an osmotic control. AMPK phosphorylation was detected by Western blot (A) and quantified using ImageQuant and normalized to the mannitol treatment group (B). β‐Actin was used as loading control. The effect of the AMPK inhibitor compound C on FGF21 increasing the NAD+ content of HG‐treated EPCs was detected using an NAD/NADH assay kit (C). The effects of inhibiting AMPK on FGF21 improving the angiogenic function of EPCs were evaluated by Matrigel tube formation assay (D), and the tube length was quantified by Image J and normalized to that of the mannitol treatment group (E). The effects of inhibiting AMPK on FGF21‐enhanced EPC migration were evaluated by scratch recovery assay (F), and the scratch recovery was quantified using Image J and normalized to that of the mannitol group (G). (H) The proposed mechanism by which FGF21 enhances the mobilization, attenuates the dysfunction of diabetic EPCs and enhances angiogenesis. The data shown in the graphs represent the means ± SD *P < 0.05 vs the mannitol group; ^#^ *P* < 0.05 vs the HG group; and ^&^ *P* < 0.05 vs the HG + FGF21 group. Bar = 100 μm

AMPK was reported to regulate the NAD^+^ content of C2C12 myocytes.^29^ Western blot results showed that HG treatment significantly repressed the phosphorylation of AMPK in the EPCs, which could be prevented by FGF21 treatment (Figure 7A,B). To confirm the importance of AMPK in this process, we further investigated the effect of blocking AMPK with its inhibitor, compound C, on the NAD^+^ content of the EPCs. The results showed that compound C abolished the effect of FGF21 in increasing NAD^+^ content under HG conditions (Figure 7C), revealing that FGF21 increased the NAD^+^ content of the EPCs mainly by activating AMPK. Moreover, compound C obviously eliminated the beneficial effect of FGF21 on the tube formation (Figure 7D,E) and migration (Figure 7F,G) capability of the HG‐treated EPCs, further confirming the critical role of the AMPK pathway in the FGF21 amelioration of EPC dysfunction induced by HG treatment.

## 4 DISCUSSION

In the present study, we found that FGF21 promotes post‐HLI angiogenesis and blood reperfusion in both T2DM and T1DM mouse models, which may be attributable to the capability of FGF21 to enhance EPC mobilization and angiogenic function. Furthermore, FGF21 directly acts on EPCs and protects EPC angiogenic function predominantly by increasing NAD^+^ content in an AMPK‐dependent manner (Figure 7H).

FGF21 exerts considerable pharmacological effects in ameliorating hyperglycaemia, dyslipidaemia and obesity as has been consistently replicated in rodent^5^, ^6^, ^8^ and monkey^30^ models of diabetes mellitus. Notably, FGF21 does not induce adverse effects such as mitogenicity, hypoglycaemia^30^ or weight gain at any dose or when overexpressed in transgenic mice.^10^ Considering its multiple metabolic benefits, several clinical trials have been registered to evaluate the therapeutic efficacy of human FGF21 analogues (LY2405319, PF‐05231023, PF‐05231023, BMS‐986036) for the treatment of T2DM, dyslipidaemia and NAFLD, and positive results have been achieved.^11^ In the present study, FGF21 obviously accelerated blood reperfusion and enhanced neovascularization in the diabetic HLI model mice (Figures 1 and 2), indicating the therapeutic potential of FGF21 in diabetic vascular complications.

In T2DM HLI model, FGF21 lowered blood glucose and induced bodyweight loss (Figure 1E,F). However, the beneficial effects of FGF21 on enhancing blood perfusion appeared earlier than its effects on blood glucose or bodyweight reduction (Figure 1B,E,F). In T1DM HLI model, FGF21 did not affect either blood glucose or bodyweight (Figure 2G,H) but still improved blood perfusion and angiogenesis in ischaemic hind limbs (Figure 2A‐F). Furthermore, FGF21 promoted EPC mobilization (Figure 3A,B) and EPC participation in ischaemic angiogenesis (Figure 3C,D). Moreover, FGF21 directly ameliorated the impaired tube formation and migration capability of EPCs treated with HG in vitro (Figure 5A‐F). These results demonstrate that FGF21 promotes EPC mobilization and preserves EPC angiogenic function, contributing greatly to the improved diabetic ischaemia angiogenesis and blood perfusion. Both EPC mobilization and EPC function are impaired under diabetic conditions.^31^ SDF‐1 is considered a principal regulator of EPC mobilization, migration and retention at angiogenic sites.^32^ Hypoxia in ischaemic tissue induces hypoxia‐inducible factor 1α (HIF‐1α) up‐regulation and thereafter enhances SDF‐1 production,^33^ and the SDF‐1 concentration gradient between ischaemic tissues and bone marrow drives EPC mobilization.^34^ However, the stability and activity of HIF‐1α induced by hypoxia were decreased under diabetic conditions,^35^ resulting in decreased SDF‐1 production and therefore impaired EPC mobilization, whereas administration of SDF‐1 into ischaemic tissues reversed the impairment to EPC mobilization and homing.^36^ In line with these findings, we found that FGF21 treatment increased circulatory SDF‐1 concentrations (Figure 3E) in T1DM mice and enhanced EPC mobilization and infiltration into ischaemic tissue (Figure 3A,B); the role of HIF1‐a stability in this process needs further investigation.

Moreover, accumulating evidence demonstrates that increased oxidative stress in EPCs contributes to impaired EPC mobilization under diabetic conditions,^37^ which can be rescued by antioxidant treatment.^38^ Similarly, our latest research showed that overexpression of the antioxidant protein metallothionein can preserve EPC mobilization in diabetic mice.^39^ Therefore, we suggest that FGF21 ameliorates oxidative stress (Figure 5G,H), which contributes to its protective effects against diabetes‐induced impairment of EPC mobilization.

EPC dysfunction also contributes to diabetes‐induced impairment in angiogenesis.^3^ NAD^+^, both a coenzyme for hydride‐transfer enzymes and a substrate for NAD^+^‐consuming enzymes, plays central roles in cellular metabolism, energy production and survival.^40^ A previous study demonstrated that NAD^+^ enhances the proangiogenic function of EPCs and improves post‐ischaemic neovascularization.^26^ Decreased NAD^+^ concentration in diabetic EPCs results in EPC dysfunction; in contrast, enhancing the NAD^+^ pool promotes the mobilization and angiogenic function of EPCs.^21^ In the current study, we found that FGF21 enhanced the NAD^+^ concentration in HG‐treated EPCs (Figure 6A), which is consistent with the research of Chau and colleagues on adipocytes,^41^ and depletion of the NAD^+^ pool eliminated the beneficial effect of FGF21 on the proangiogenic function of EPCs (Figure 6F,G). Moreover, NAD^+^ deficiency also contributes to vascular ageing.^42^, ^43^ Therefore, the FGF21 reversal of EPC senescence induced by HG (Figure 5E,F) may also depend on its capability to elevate the NAD^+^ concentration. These facts demonstrate a crucial role of NAD^+^ in the FGF21 protection of EPCs against diabetes‐ or HG‐induced dysfunction.

NAD^+^ content is largely regulated by NAMPT, the rate‐limiting enzyme for NAD^+^ biosynthesis,^26^ and NAD^+^‐consuming enzymes, such as PARP and CD38.^40^ NAMPT promotes the angiogenic function of EPCs and improves ischaemic angiogenesis,^26^ whereas PARP^28^ and CD38^27^ trigger EPC dysfunction. However, the expression levels of NAMPT, PARP or CD38 in EPCs were unchanged in the present study (Figure S4), which may be due to differences in the cell models among studies. Canto and colleagues^29^ found that activation of AMPK can rapidly increase the NAD^+^ content in C2C12 cells, and this effect is not affected by NAMPT inhibition.^29^ In the present study, AMPK phosphorylation was impaired by HG and rescued by FGF21 treatment (Figure 7A,B). Inhibiting AMPK abrogated the increase in NAD^+^ induced by FGF21 (Figure 7C), which indicates that FGF21 elevates NAD^+^ content in HG‐treated EPCs by activating AMPK. AMPK has been reported to mediate the beneficial effects of metformin,^44^ resveratrol^45^ and fenofibrate^46^ on the function of endothelial cells or EPCs under diabetic conditions. In the present study, we also found that blocking AMPK impaired the protective effect of FGF21 against EPC dysfunction induced by HG (Figure 7D‐G), which is consistent with the findings on endothelial cells obtained by Ying and colleagues.^47^

Sirtuins are a family of NAD^+^‐dependent deacetylases with remarkable importance in cell stress resistance.^48^ Among sirtuins, only Sirt1 was found to be relevant to NAD^+^‐improved diabetic EPC function in the present study (Figure S3), although its expression was unchanged (Figure S5A,B). A recent study found that Sirt1 is a key regulator of vascular endothelial homeostasis because it adjusts the activity of a variety of substrates, including FOXOs, NF‐κB, NOX, SOD and eNOs, via deacetylation.^49^ Sirt1 mediates the protective effect of metformin on endothelial cells against HG‐induced premature senescence and apoptosis by deacetylating FOXO1 and p53/p21.^50^ Similarly, Sirt1 can protect against oxidative stress‐induced EPC apoptosis by reducing FOXO3a acetylation levels and increasing its degradation.^51^ Moreover, SIRT1 inhibits NF‐κB signalling induced by HG by directly deacetylating the p65 subunit of the NF‐κB complex.^52^ Therefore, we deduce that NAD^+^ improves diabetic EPC function predominantly through Sirt1.

The present study demonstrates that FGF21 promotes ischaemic angiogenesis and blood perfusion under diabetic conditions and improves the angiogenic capability of EPCs by activating the AMPK/NAD^+^ pathway, which provides experimental evidence for the potential application of FGF21 in the therapy of diabetic vascular complications.

## CONFLICT OF INTEREST

The authors confirm that there are no conflicts of interest.

## AUTHOR CONTRIBUTION

**Qiaoxia Dai:** investigation (lead). **Xia Fan:** investigation (equal). **Xue Meng:** investigation (supporting). **Shiyue Sun:** Investigation (supporting). **Yue Su:** investigation (supporting). **Xiao Ling:** investigation (supporting). **Xiangjuan Chen:** investigation (supporting). **Kai Wang:** investigation (supporting). **Xiaozhen Dai:** investigation (supporting). **Chi Zhang:** investigation (supporting). **Da Sun:** investigation (supporting). **Guigui Zhang:** investigation (supporting). **Chunjie Gu:** investigation (supporting). **Hui Chen:** investigation (supporting). **Junhong He:** investigation (supporting). **Haiqi Hu:** investigation (supporting). **Lechu Yu:** investigation (supporting). **Xiaohong Pan:** investigation (supporting). **Yi Tan:** writing‐review & editing (equal). **Xiaoqing Yan:** supervision (lead); writing‐original draft (lead).

## Supporting information

Supplementary Material

## Data Availability

The datasets generated and/or analysed during the current study are available from the corresponding author upon reasonable request.
