# Supplementary material for: FGF21 promotes ischaemic angiogenesis and endothelial progenitor cells function under diabetic conditions in an AMPK/NAD+‐dependent manner
Source: J Cell Mol Med. 2021 Feb 17;25(6):3091–102. doi: 10.1111/jcmm.16369 (PMC7957202; doi:10.1111/jcmm.16369)
Supplement: Supplementary file 1 — Supplementary Material [file JCMM-25-3091-s001.pdf]

Supplement

Table S1

siRNA information		
Target Gene	sense（5'-3'）	antisense（5'-3'）
Sirt1	CCAAGCAGCUAAGAGUAAUTT	AUUACUCUUAGCUGCUUGGTT
Sirt2	CCAUCUGUCACUACUUCAUTT	AUGAAGUAGUGACAGAUGGTT
Sirt3	CCAGUGGCAUUCCAGACUUTT	AAGUCUGGAAUGCCACUGGTT
Sirt4	GCGUGUCUGAAACUGAAUUTT	AAUUCAGUUUCAGACACGCTT
Sirt5	CUCGCCCACUGUGAUUUAUTT	AUAAAUCACAGUGGGCGAGTT
Sirt6	GCCAAGUGUAAGACGCAGUTT	ACUGCGUCUUACACUUGGCTT
Sirt7	GCCAAAUACUUGGUCGUCUTT	AGACGACCAAGUAUUUGGCTT
NAMPT	GCAGAACACAGUACCAUAATT	UUAUGGUACUGUGUUCUGCTT

Table S2

Primer information	
FGFR1-F	GTTCTTCTGGTTCGGCCATC
FGFR1-R	GGTTGGGTTTGTCCTTGTCC
FGFR2-F	GTTTAAGCAGGAGCATCGCA
FGFR2-R	AACATCCAGGTGGTACGTGT
FGFR3-F	GGAGAACAAGTTTGGCAGCA
FGFR3-R	CGTCACTGTACACCTTGCAG
FGFR4-F	AAGCACATCGTCATCAACGG
FGFR4-R	CAGGCAGACTGGTAGGAGAG
NAMPT-F	GCCTTCGGTTCTGGTGGAGG
NAMPT-R	TCCCTGCTGGCGTCCTATGT
Sirt1-F	GACTGGACTCCAAGGCCACG
Sirt1-R	ACAGACACCCCAGCTCCAGT
Sirt2-F	TCTGCTGGACGAGCTGACCT
Sirt2-R	TGGAGAGCGAAAGTCGGGGA
Sirt3-F	ACGATCTCCCGTACCCCGAG
Sirt3-R	TTGGCAGACTGTGCAGGTGG
Sirt4-F	CCCTGCACACTGGGCTTTGA
Sirt4-R	GGACCCTGTCCATGCATCCG
Sirt5-F	CTTCCCCGGTGTGAAGAGGC
Sirt5-R	TCGTAGCTGGGGTGGTCTCC
Sirt6-F	GGGAGAGCTGAGGGACACCA
Sirt6-R	CTTGGTGGGCTGCAGGTTGA
Sirt7-F	GTGAACCTGCAGTGGACCCC
Sirt7-R	TTGTGCGTTTTGTGCAGCCC
β-Actin-F	GAGCTACGAGCTGCCTGACG
β-Actin-R	TGCCAGGGCAGTGATCTCCT

Fig. S1

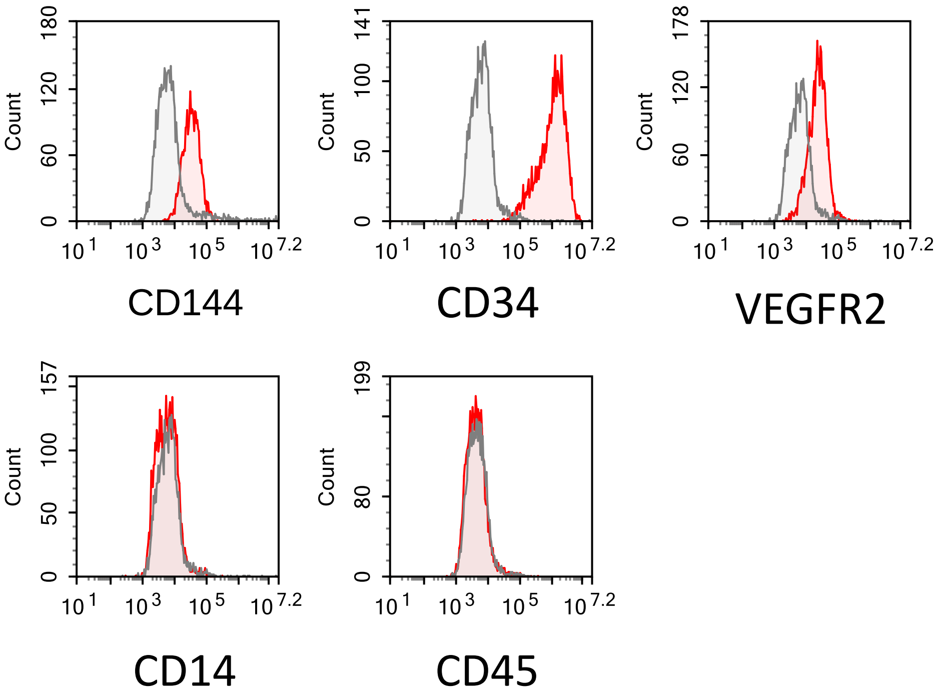

Fig. S2

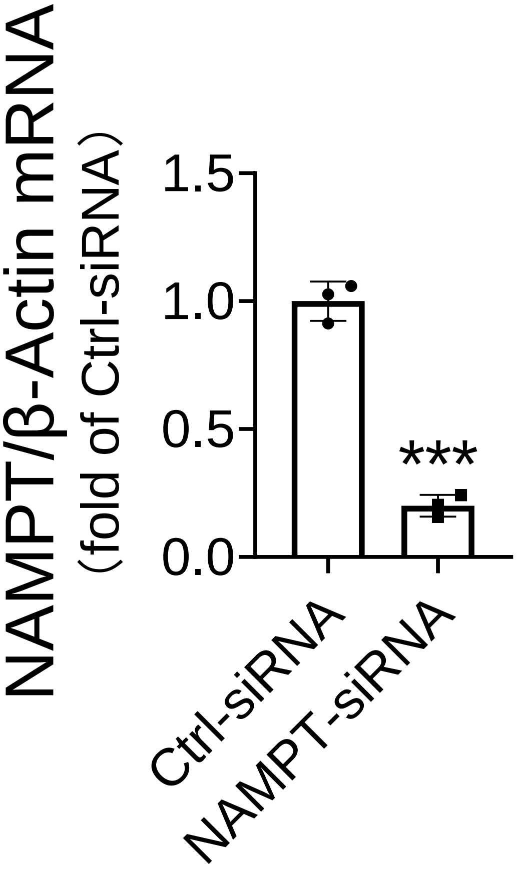
NAMPT/β-Actin mRNA
（fold of Ctrl-siRNA）
1.5
1.0
0.5
0.0
***
Ctrl-siRNA
NAMPT-siRNA

Fig. S3

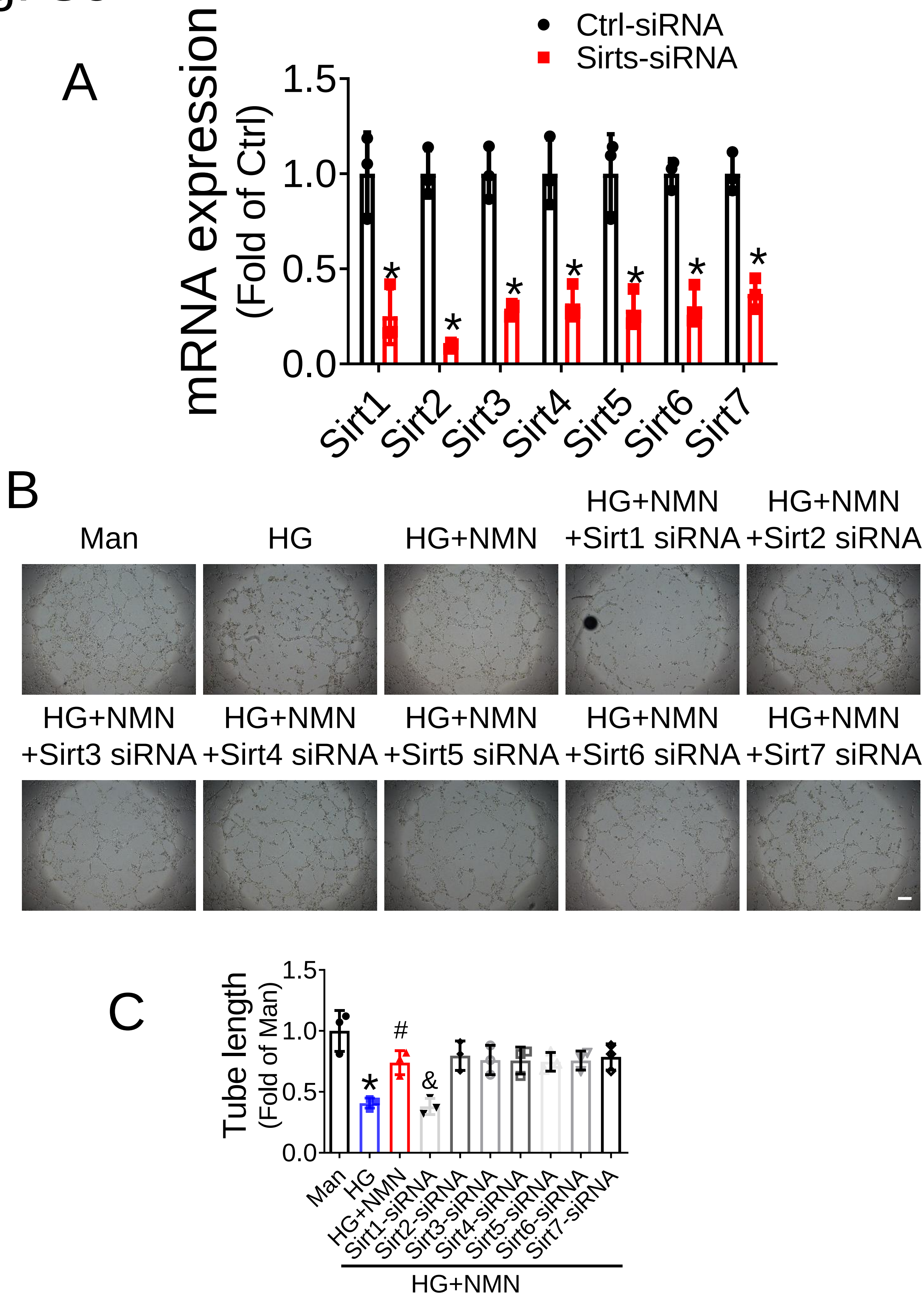
A
mRNA expression
(Fold of Ctrl)
Ctrl-siRNA
Sirts-siRNA
1.5
1.0
0.5
0.0
Sirt1
Sirt2
Sirt3
Sirt4
Sirt5
Sirt6
Sirt7
B
Man
HG
HG+NMN
HG+NMN +Sirt1 siRNA
HG+NMN +Sirt2 siRNA
HG+NMN +Sirt3 siRNA
HG+NMN +Sirt4 siRNA
HG+NMN +Sirt5 siRNA
HG+NMN +Sirt6 siRNA
HG+NMN +Sirt7 siRNA
C
Tube length
(Fold of Man)
1.5
1.0
0.5
0.0
#
&
Man
HG
HG+NMN
Sirt1-siRNA
Sirt2-siRNA
Sirt3-siRNA
Sirt4-siRNA
Sirt5-siRNA
Sirt6-siRNA
Sirt7-siRNA
HG+NMN

Fig. S4

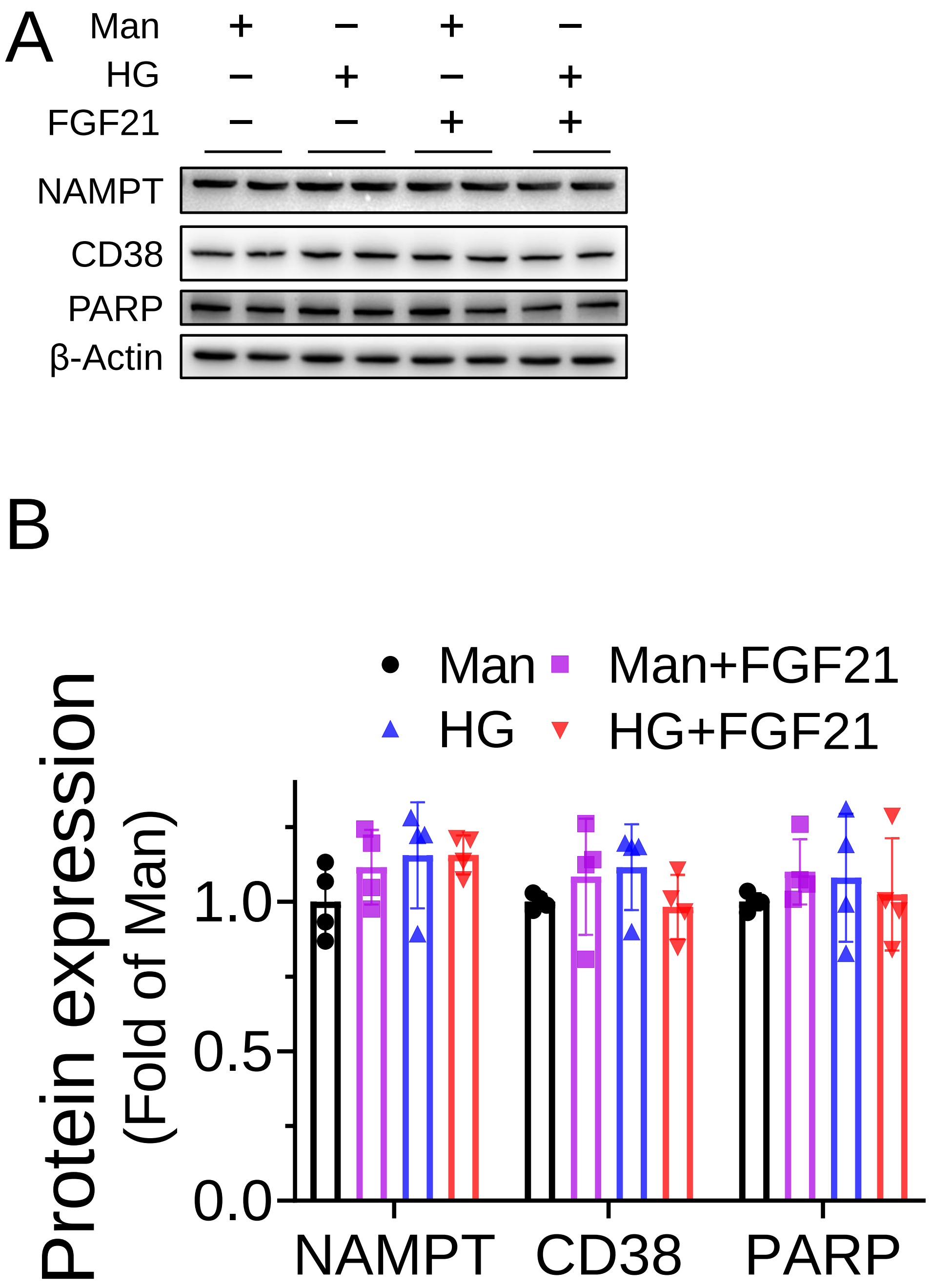
A
Man + − + −
HG − + − +
FGF21 − − + +
NAMPT
CD38
PARP
β-Actin
B
Man
Man+FGF21
HG
HG+FGF21
Protein expression (Fold of Man)
0.0
0.5
1.0
NAMPT
CD38
PARP

Fig. S5

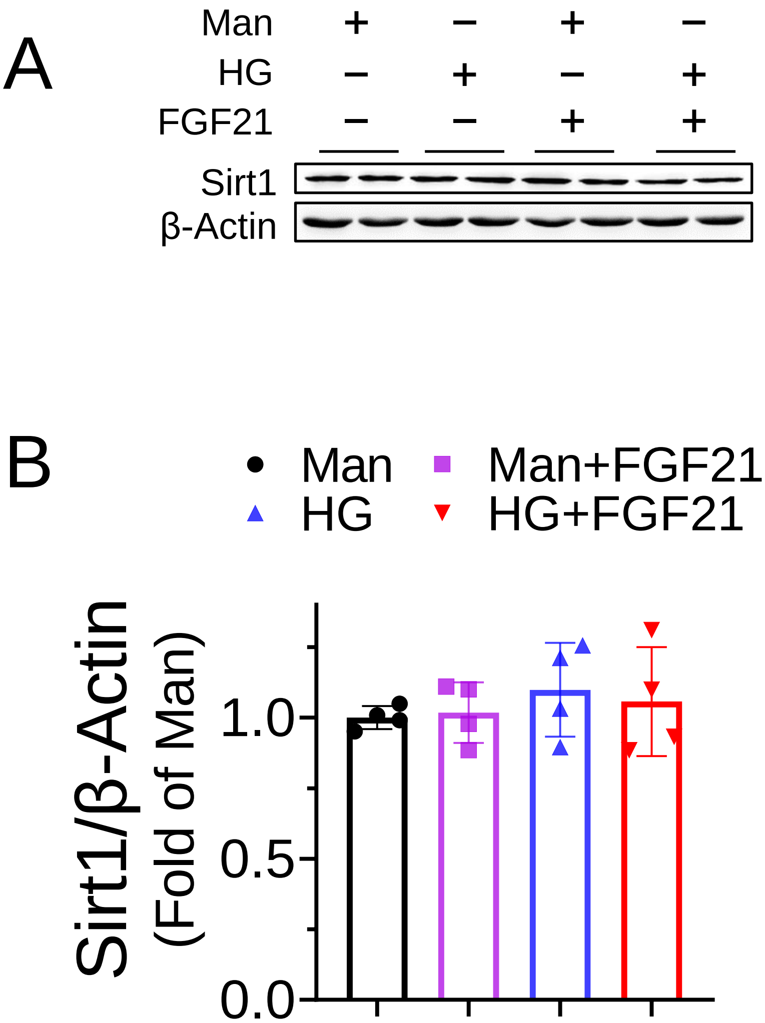
A
Man + − + −
HG − + − +
FGF21 − − + +
Sirt1
β-Actin
B
Man
HG
Man+FGF21
HG+FGF21
Sirt1/β-Actin
(Fold of Man)
1.0
0.5
0.0
